# Using Rapid Analyte Measurement Platform (RAMP) as a Tool for an Early Warning System Assessing West Nile Virus Epidemiological Risk in Bucharest, Romania

**DOI:** 10.3390/tropicalmed7110327

**Published:** 2022-10-23

**Authors:** Florian Liviu Prioteasa, Georgiana Victorița Tiron, Sorin Dinu, Elena Fălcuţă

**Affiliations:** 1 Medical Entomology Laboratory, Cantacuzino National Military Medical Institute for Research and Development, 050096 Bucharest, Romania; 2 Vector-Borne Infections Laboratory—Cantacuzino National Military Medical Institute for Research and Development, 050096 Bucharest, Romania; 3 Molecular Epidemiology for Communicable Diseases Laboratory, Cantacuzino National Military Medical Institute for Research and Development, 050096 Bucharest, Romania

**Keywords:** West Nile virus, rapid screening, mosquito pools, real-time RT-PCR, RAMP, false positive

## Abstract

West Nile virus (WNV) is the most widely spread arbovirus in the world. Early detection of this virus in mosquito populations is essential for implementing rapid vector control measures to prevent outbreaks. Real-time reverse transcription polymerase chain reaction (real-time RT-PCR) is a powerful tool for the detection of WNV in mosquito pools, but it is a time- and resource-consuming assay. We used a Rapid Analyte Measurement Platform (RAMP) assay in a vector surveillance program for rapid detection of WNV in mosquitoes collected in Bucharest city, Romania, in 2021. The positive mosquito pools were tested for confirmation with real-time RT-PCR. Three out of the 24 RAMP assay positive pools were not confirmed by real-time RT-PCR. We consider that RAMP assay can be used as a fast and reliable method for the screening of WNV presence in mosquito pools, but we recommend that samples with values ranging from 30 to 100 RAMP units should fall in a grey zone and should be considered for real-time RT-PCR confirmation.

## 1. Introduction

West Nile virus (WNV) is a flavivirus maintained in enzootic cycles between birds and ornithophilic mosquito vectors. The virus is transmitted tangentially to mammals, including to humans, through mosquito bites. Mammals are considered dead-end hosts since their low level of viremia is usually not sufficient to infect mosquitoes. Most human infection cases are inapparent or display mild symptoms; however around 1% of them develop into severe neurological disease. The high genetic diversity of WNV is reflected in the existence of up to nine lineages with distinct evolution, pathogenicity, and geographic distribution [1,2,3]. However, only lineage 1 and lineage 2 WNV isolates are considered pathogenic for human and caused severe outbreaks across Europe, posing serious public health issues. Lineage 1 WNV isolates were responsible for the first outbreaks in Europe (beginning 1960s), whereas the emergence of lineage 2 begun in 2004 in Hungary with isolates belonging to central/southern European clade and continued with a subsequent introduction of eastern European clade isolates in Russia (2007) and Romania (2010) [4,5,6,7,8,9].

The first documented outbreak of WNV human infection in Romania occurred in 1996 in the southern part of the country, when most of the 393 confirmed cases represented neurological infections [10] and was caused by a viral isolate belonging to lineage 1 [11]. In the subsequent years, increased numbers of cases or outbreaks were also recorded [12]. *Culex pipiens* sensu lato (*Cx. pipiens* s.l.) mosquitoes were identified as main vectors of WNV in Romania [11,13] and it was shown that the two morphologically indistinguishable biotypes of *Cx. pipiens* (*Cx. pipiens pipiens* and *Cx. pipiens molestus*) play different roles in WNV transmission cycle [14]. The southern part of the country, including Bucharest city, is susceptible to the introduction of WNV, the circulation of lineage 1 and lineage 2 strains being documented so far [11,13,15]. Strains belonging to lineage 2 central/southern European clade and eastern European clade cocirculated and the first one replaced the latter [15].

Early detection of the virus in mosquito populations is essential for implementing targeted vector control measures to prevent outbreaks. Since 2019, we have started the implementation of an early warning system to detect WNV risk areas in Bucharest city. This warning system is based on the identification of the virus in mosquito populations followed by notification of the local and national authorities responsible for vector control, in order to take actions for preventing the onset of WNV human infection cases. For the first two years of our study we employed real-time RT-PCR for the detection of WNV in mosquito pools. Real-time RT-PCR is the most used method for WNV detection in human and mosquito samples; however the technique is laborious and requires trained personnel, expensive facilities and reagents. Additionally, a time delay between the mosquito collection, diagnosis, and reporting the results may be generated by the shipment of the samples to a testing facility. Furthermore, due to the vast genetic diversity of WNV real-time RT-PCR tests must be tuned to detect as many as possible viral isolates. Assays for detection of all known lineages were proposed [16], but methods have to be regularly checked and updated in order to detect emerging strains and isolates [17]. Given the time and financial limitations, for the third year of our study we decided to also use Rapid Analyte Measurement Platform (RAMP) with RAMP^®^ WNV test (Response Biomedical Corporation, Burnaby, British Columbia, Canada). This antigen-based assay delivers fast results (2 h) and can be used on site with minimum equipment and hands-on time. The test has been also evaluated in previous studies and the results were compared in terms of specificity and sensitivity with real-time RT-PCR, cell culture, or other antigen-based methods [18,19,20].

In our study, the mosquito pools found positive for WNV by RAMP assay were also tested by real-time RT-PCR for confirmation and comparison of the two methods.

## 2. Materials and Methods

### 2.1. Mosquito Collection and Processing

Fifteen investigation sites were chosen in different locations of Bucharest, the capital city of Romania. The city is located in southeastern of the country, in Danube Plain, and is crossed by Dâmboviţa river. The investigation sites represent a wide range of urban areas such as green areas, aquatic areas, residential areas with blocks of flats, residential areas with individual dwellings, and mixed residential areas. Another site located in Ilfov county, which belongs to the Bucharest metropolitan area, was investigated exclusively in 2019. Due to the large volume of work, in the following two years of study, the investigations took place only in the city area. Mosquitoes were collected between 2019 and 2021, each year from the beginning of June until late September, using CDC gravid traps. These traps preferentially attract pregnant mosquito females that have taken a blood meal, thus increasing the chances of finding positive samples. The traps were left to operate continuously and the batteries were replaced weekly while the bags with the collected material were changed usually every 2–3 days. Mosquitoes were identified to the species level using morphological dichotomous keys described by Becker et al. [21]. Following identification, female mosquitoes were grouped into pools of 20–50 individuals, according to the date and site of collection, and kept at −20 °C until screening for WNV.

### 2.2. WNV Detection by Real-Time RT-PCR

Mosquito pools were homogenized in 1 mL of PBS solution (pH 7.2) supplemented 20% with fetal calf serum, 200 µg/mL streptomycin, 200 U/mL penicillin, 1 µg/mL amphotericin B, using Sartorius Mikro-Dismembrator U laboratory bead mill (2000 rpm, 3 min). The homogenate was centrifuged (14,000 rpm, 4 °C) and the supernatant was collected. RNA was extracted from 140 µL supernatant using QIAamp Viral RNA Mini Kit (Qiagen, Hilden, Germany) and eluted in 50 µL of AVE buffer provided in the kit. RNA extraction was carried out in a BSL-2 facility. After extraction, 10 µL of RNA solution were used for screening the presence of WNV genome by one-step real-time RT-PCR using a commercial kit (West Nile Virus Real-TM, Sacace Biotechnologies, Como, Italy) on a QuantStudio 5 Real-Time PCR System (Applied Biosystems, Waltham, MA, USA).

### 2.3. WNV Detection by RAMP Assay

RAMP^®^ WNV (Response Biomedical Corporation, Burnaby, BC, Canada) is a semiquantitative immunochromatographic screening test used for rapid diagnose of WNV in mosquito pools and bird throat swabs. The kit uses fluorescent-dyed particles coated with anti-WNV antibodies which bind to WNV, if present in the sample. Mosquito pools are homogenized in a provided buffer solution, mixed with the fluorescent antibodies, and inserted to migrate in a cartridge which contains a strip. WNV particles conjugated with fluorescent antibodies are captured at the test zone of the strip, while the free antibodies are bound at the control zone. A volume of 1000 µL RAMP buffer solution and two cooper beads were added to each mosquito pool which were further homogenized by vortexing and subjected to centrifugation for 5 min at 5000 rpm. One hundred twenty µL of the supernatant were transferred to a new 0.5 mL tube provided in the kit. The rest of the supernatant was discarded and only pellets were kept for real-time RT-PCR confirmation tests. Seventy µL of supernatant were mixed well with the fluorescent labeled antibodies and inserted to migrate at least 90 min in the RAMP cartridge. RAMP reader measures the fluorescence intensity at both test and control zones. The ratio is given in RAMP units with values ranging between 0 and 640. According to the manufacturer’s instructions a mosquito pool displaying a value > 30 units is considered positive for WNV. RAMP assay was performed in a BSL-2 facility.

## 3. Results

In 2019 and 2020, mosquito pools were tested by the real-time RT-PCR technique only. A total number of 223 pools comprising 8060 *Cx. pipiens* s.l. females were tested in 2019. Twenty-three pools were positive for WNV, fifteen from Bucharest city and eight from the site located in Ilfov county. Mosquitoes from the positive pools from the city were collected in four out of the fifteen investigated sites, minimum infection rate (i.e., [number of positive pools / total specimens tested] × 1000) ranging between 1.35 and 5.99‰. Mosquitoes from the first positive pool were collected on August 2 but, unfortunately, the time delay between the mosquito collection and real-time RT-PCR test was too long, and the confirmation of the first mosquito positive samples coincided with the recording of the first WNV human cases. Therefore, the early warning system did not achieve its intended goal. A total of eighteen cases of WNV human infection were recorded in Bucharest city that year. In 2020, we tried to minimize the time delay between the mosquito collection and the real-time RT-PCR diagnostic. A total number of 18,156 females of *Cx. pipiens* s.l. (out of >43,000 collected mosquitoes) were grouped into 486 pools and tested. Additionally, six pools comprising 231 females of *Aedes albopictus* were tested and found negative for WNV. Fourteen pools of *Cx. pipiens* collected in eight sites were positive, the first one being collected on 10 July. The minimum infection rate ranged between 0.51 and 4.5‰. The authorities were informed immediately after the identification of each positive sample and vector control measures were implemented in each risk area. Only one case of human infection with WNV was recorded in the city. Our goal was achieved in 2020, but the costs and work effort were significant.

During 2021 season, 18,326 mosquitoes (14,970 females and 3356 males) were collected in the fifteen investigated sites. Ten species were identified, with *Cx. pipiens* and *Aedes albopictus* being by far the most abundant. A total number of 219 mosquito pools were tested for WNV presence by RAMP assay (210 pools consisting of 8878 *Cx. pipiens* females and nine pools consisting of 433 *Aedes albopictus* females). Twenty-four pools of *Cx. pipiens* collected in four sites were found positive for WNV by RAMP assay, the authorities being informed immediately. All pools of *Aedes albopictus* females were negative for WNV. Only three human cases of WNV infection were recorded in Bucharest in 2021. The RAMP positive pools were subsequently tested by real-time RT-PCR which confirmed 21 out of 24 samples (Table 1). The minimum infection rate for 2021 was calculated using real-time RT-PCR data which ranged between 1.67 and 6.4‰.

## 4. Discussion

In our hands, RAMP assay used for the screening of WNV in mosquito pools worked well, generating similar results with the ones obtained in the first two years of the study, when only real-time RT-PCR test was used. Therefore, by using RAMP assay we were able to produce results in a timely manner and to notify the national public health authorities and the local authorities responsible for vector management before the onset of the first human cases of WNV infection.

Twenty-one out of the 24 samples found WNV positive by the RAMP assay in the third year of study were further confirmed by real-time RT-PCR. The three false positive mosquito pools displayed values for RAMP units between 35.2 and 85.7. It is worth mentioning that other three pools with similar values for RAMP units were confirmed by real-time RT-PCR. Previous studies evaluated the usefulness and reliability of the RAMP assay as a WNV testing tool for mosquito pool samples, both in field and laboratory conditions, and found contradictory results in terms of specificity and sensitivity. A study conducted in United States and investigating the false positive rates of RAMP assay used in mosquito control programs included as the causes of false positive results: improper sample preparation, RAMP testing, storage, and shipping; improper RT-PCR testing and variation among WNV primer/probe sets; variation in mosquito species and/or physiological stages causing variation in RAMP and RT-PCR results; and variation in the sensitivity of the RAMP kit. When the authors applied a cut-off value of 80 RAMP units, the number of false positive samples decreased whereas the number of false negative samples increased [22]. As also stated by the manufacturer, factors such as blood from engorged mosquito females can lead to false positive results in RAMP assay.

In our sample series, RAMP unit values did not always correlate with Ct values. Some samples displayed low Ct values and low RAMP unit values indicating that other factors, and not the quantity of the virus present in the sample, affected the sensitivity of the RAMP test. On the other hand, some samples displayed higher Ct values and high RAMP unit values, suggesting inhibition in real-time RT-PCR test or non-specificity of the RAMP. The inhibitory effect of the RAMP buffer over the real-time PCR was previously shown [20] therefore we did not use the supernatant obtained by homogenization with RAMP buffer, instead we used the pelleted material as an input for RNA extraction. Low correlation between the Ct values and RAMP results was also observed by Kesavaraju et al. [22] and attributed to the different efficacy of primer/probe sets used for detection of WNV. The authors concluded that primer/probe sets used for comparison to RAMP could greatly impact the interpretation of RAMP efficacy. However, another field study analyzing *Culex* sp. mosquitoes found 100% specificity and 94% sensitivity for RAMP assay when using the threshold recommended by the manufacturer (>30 RAMP units for a positive sample) and comparing with real-time RT-PCR [19]. When comparing with another antigen-based test, also designed to be used in field studies, RAMP proved to have increased sensitivity (94% versus 65% positive samples correctly identified) [23].

Biosafety concerns regarding the potential hazard of homogenization of mosquito samples outside of a BSL-3 facility (such as in field studies) were addressed in a recent study and alternate sample homogenization protocol using Triton X-100 detergent that ensures complete WNV inactivation without compromising the performance of the RAMP assay was proposed [24].

Kesavaraju et al. [22] concluded that different laboratories testing mosquitoes use different cut-off values and that increasing the cut-off value did not always result in reducing the false positive rate, thereby strengthening the argument that the underlying cause for such variation needs to be investigated. On the other hand, Burkhalter et al. [20] recommended considering a cut-off value of 50 units for RAMP positive pools with no real-time RT-PCR confirmation to maximize speed, efficiency, and economy of the RAMP assay. Alternatively, the study recommends a more conservative approach with the implementation of a grey zone ranging between 50 and 100 RAMP units, pools scoring within the grey zone can be submitted for real-time RT-PCR confirmation.

Based on our results, we consider that RAMP assay can be used as a rapid and reliable method for screening the presence of WNV in mosquito pools. We consider that 30 units should be the cut-off value for the RAMP positive samples and the grey zone for which we recommend real-time RT-PCR confirmation should be the 30–100 units interval. However, our study is ongoing and as we collect more data, we will be able to confirm or to adjust the proposed interval for the grey zone.

## 5. Conclusions

Although it requires the consideration of a grey zone, the RAMP assay is a reliable, fast, and inexpensive method, suitable for field medical entomology studies in which rapid identification of WNV in mosquito vector populations is needed.

## Figures and Tables

**Table 1:** Real-time RT-PCR results for *Culex pipiens* female pools testing positive for WNV in RAMP assay.

Pool No.	Individuals in Pool	Date of Collection	RAMP Assay (Units)	PCR Result/Ct Value
R21-35	45	July 12	85.6	Positive/33.18
R21-42	34	July 14	85.7	Negative/NA *
R21-52	45	July 19	35.9	Positive/27.2
R21-83	41	July 26	219	Positive/17.8
R21-91	47	July 19	45.8	Negative/NA
R21-93	38	July 25	35.2	Negative/NA
R21-104	50	July 26	640	Positive/17.8
R21-107	45	July 26	120.8	Positive/24.32
R21-121	40	July 28	37.8	Positive/27
R21-151	40	August 6	231.3	Positive/31.02
R21-156	40	August 9	186	Positive/21.63
R21-161	50	August 10	640	Positive/24.44
R21-164	50	August 10	640	Positive/21.74
R21-177	33	August 13	191	Positive/31.08
R21-179	32	August 13	640	Positive/27.28
R21-184	47	August 21	640	Positive/26.34
R21-198	50	August 18	640	Positive/29.62
R21-199	50	August 18	640	Positive/27.62
R21-200	48	August 18	640	Positive/27.36
R21-202	50	August 24	640	Positive/22.94
R21-204	50	August 24	640	Positive/26.35
R21-206	36	August 25	616	Positive/28.09
R21-209	45	August 24	484.8	Positive/26.83
R21-222	41	August 30	91.2	Positive/23.98

* NA: not applicable.

## Data Availability

Not applicable.
